# Possible Evidence for a New Form of Liquid Buried in the Surface Tension of Supercooled Water

**DOI:** 10.1038/srep33284

**Published:** 2016-09-12

**Authors:** T. Ryan Rogers, Kai-Yang Leong, Feng Wang

**Affiliations:** 1 Department of Chemistry and Biochemistry, University of Arkansas, Fayetteville, AR 72701, USA.

## Abstract

Contrary to the historical data, several recent experiments indicate that the surface tension of supercooled water follows a smooth extrapolation of the IAPWS equation in the supercooled regime. It can be seen, however, that a small deviation from the IAPWS equation is present in the recent experimental measurements. It is shown with simulations using the WAIL water potential that the small deviation in the experimental data is consistent with the tail of an exponential growth in surface tension as temperature decreases. The emergence temperature, *T*_*e*_, of a substantial deviation from the IAPWS equation is shown to be 227 K for the WAIL water and 235 K for real water. Since the 227 K *T*_*e*_ is close to the Widom line in WAIL water, we argue that real water at 235 K approaches a similar crossover line at one atmospheric pressure.

Recently, the surface tension of supercooled water has been measured down to approximately −25 °C by two independent groups using three different experimental setups^1^,^2^. The new measurements are consistent with each other and show a gradual increase of the surface tension at lower temperature that closely follows the IAPWS correlation of ordinary water. The new data are argued to be more reliable than the previous experimental data^3^, such as those obtained by Mohler^4^ in 1895 and by Hacker^5^ in 1951. The historical data show a kink at −9 °C^5^, which could be interpreted as evidence for a second inflection point. The existence of such an anomaly was viewed as suggesting the existence of a new form of metastable water in the supercooled regime^6^,^7^. However, the new surface tension data resemble a smooth extrapolation of the IAPWS correlation equation and give no clear evidence of any anomaly.

Figure 1 shows the deviation of the recent experimental surface tension of supercooled water from the IAPWS correlation. It is interesting to note that the deviation is always positive at lower temperatures and increases as temperature decreases. The error bar used by Hruby *et al.*^1^,^2^ is shown as *σ*_*Hru*_ and the standard error of the mean calculated assuming the five sets of measurements are independent is shown as *σ*_*ind*_. The observed deviation of surface tension from the IAPWS equation at the lowest temperature is comparable to *σ*_*Hru*_ and significantly larger than *σ*_*ind*_. Although the five sets of data were obtained in two different years and with two different methods, it is indeed still possible that the five sets of data are correlated, which will result in the real error bar to be larger than *σ*_*ind*_. At the same time, it is difficult to rule out the possibility that the deviation is not simply due to experimental errors and a systematic trend in water surface tension is emerging as temperature drops.

## Methods

In order to further understand the deviation, molecular dynamics simulations were performed with the Water potential from Adaptive force matching for Ice and Liquid (WAIL)^8^ to determine if similar deviations can be observed. The WAIL potential was developed by an iterative force matching procedure^9^,^10^ to map a coupled-cluster quality potential energy surface^11^ for ice and water. Without fitting to any experimental properties, the WAIL potential predicts an ice-Ih melting temperature of 270 K and showed evidence of a liquid-liquid phase transition (LLPT) in supercooled water^12^ with a critical point at approximately 50 MPa and 207 K^12^. A strong-to-fragile transition^13^,^14^ has also been shown in the WAIL water with the high density liquid showing a fragile behavior and the low density liquid being strong^15^.

In this study, the surface tension (*γ*) of WAIL water was calculated from 213 to 298 K. The measurement was performed with the mechanical method^16^ through the equation


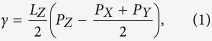


where *L*_*Z*_ is the Z dimension of the box and the factor of 2 on the left is due to the presence of two liquid-vapor interfaces in a slab. The water slab contains 2139 molecules and is continuous in the X-Y plane. The X and Y dimensions were held at 4.0 nm with the Z dimension being 10.0 nm. This allows a vacuum region of approximately 6.0 nm. In order to use a 1 fs time-step, the hydrogen isotope mass was chosen to be 3.016 g·mol^−1^, which is that of Tritium. This choice of heavier isotope should not influence the surface tension in a Newtonian MD simulation. The van der Waals interactions were truncated beyond 0.9 nm and the long range electrostatics was treated with the particle-mesh Ewald method^17^,^18^. Proper convergence of surface tension is challenging especially at lower temperatures. A total of 109.4 μs of simulations was performed in supercooled water to reduce the error bar to an acceptable value. The total length of simulations at each temperature varies from more than 20 μs at 213 K to around 1 μs above 268 K.

## Results and Discussion

The surface tension of supercooled water modeled with the WAIL potential is reported in Table 1 in the supplementary information and plotted in Fig. 2(a). From 243 to 298 K, *γ* of WAIL water shows a good fit to the IAPWS equation,


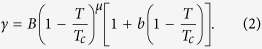


Since the critical temperature *T*_*c*_ of WAIL water has been estimated to be 711 K^19^, the IAPWS equation was fit with the previously determined *T*_*c*_ and a critical exponent (*μ*) of 11/9^20^. The fitted parameters are summarized in Table 1. The good agreement of WAIL surface tension to the IAPWS fit above −30 °C is consistent with the recent experiments^1^,^2^, where a good agreement was observed down to −25 °C.

Below 243 K, it is clear that surface tension of WAIL water no longer follows the IAPWS equation. The deviation is always positive and grows aggressively at lower temperatures. As shown in Fig. 2(b), the deviation of the WAIL surface tension from the IAPWS equation closely follows a straight line in the semi-log plot. This indicates the deviation is approximately exponential for the WAIL model down to 213 K. In order to capture such an exponential divergence, the surface tension of WAIL water from 243 to 298 K was fitted using the following equation,


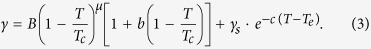


Although the exponential term has three parameters, *γ*_*s*_, *c*, and *T*_*e*_, only two of the parameters are independent. We choose *γ*_*s*_ to be 1 mN·m^−1^. When the deviation between *γ* and the IAPWS correlation is larger than *γ*_*s*_, we will then consider the deviation substantial. The temperature at which the deviation becomes substantial will be referred to as the emergence temperature, *T*_*e*_, of new physics in supercooled water. Since WAIL water has two liquid phases, we believe *T*_*e*_ represents the temperature at which the contribution from the low density liquid (LDL) to the surface tension becomes substantial. We emphasize that, although WAIL has two liquid phases, at the simulation pressure of 0.1 MPa, water is in the one phase regime and will never phase separate. The molar fraction of the LDL form will increase and starts to dominate as temperature decreases.

The fitting to equation (3), which will be referred to as the IAPWS-E equation, was performed also with a *T*_*c*_ of 711 K and a *μ* of 11/9. The resulting parameters are summarized in Table 1. The fit is plotted in Fig. 2(c). Clearly the IAPWS-E equation gives an excellent fit to the surface tension of water down to 213 K. The fit gives a *T*_*e*_ of 227 K, which is close to the Widom line^21^ temperature of 224 K at 0.1 MPa for the model^12^.

Since the WAIL water model shows an exponential increase in surface tension as temperature decreases, it is intriguing to verify if the small deviation observed experimentally at lower temperature is also consistent with an exponential component. We thus fit the IAPWS-E equation to the experimental data. We used the five sets of experimental data published by Hruby in two different publications in 2014 and 2015. The 2014 dataset was measured using the capillary rise method. Two of the 2015 datasets were measured with the counter-pressure method, and the other two 2015 datasets were measured using the capillary rise method. We combined the two datasets measured with each method into a group and fit them together. This was done since each dataset has gaps in the temperatures covered but otherwise closely follows the same trend.

**Figure 1:**
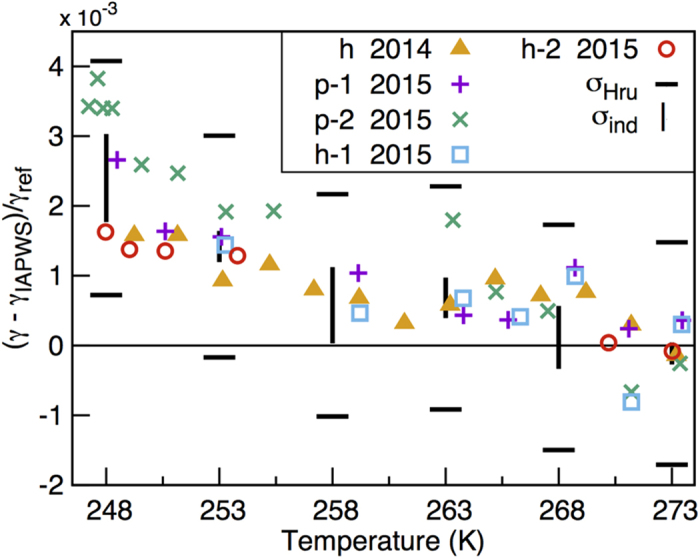
Relative deviation of experimental surface tension of water from the IAPWS-equation in the supercooled regime. All data sets labeled “2015” were taken from ref. 2, where they were listed with the same labels: h-1, h-2, p-1, and p-2. The “h 2014” data set was taken from ref. 1, where it was referred to as the “Prague set.” All “h” sets were obtained using the capillary rise method, which is also referred to as the height method^1^,^2^. All “p” sets were obtained using the counter-pressure method^2^. The horizontal bars is the error bar (*σ*_*Hru*_) published by Hruby^1^,^2^ and the vertical lines is the error bar (*σ*_*ind*_) calculated assuming the five datasets are independent. The agreement between the five sets of data is significantly better than the deviations from the IAPWS correlation, suggesting a systematic deviation arising as temperature decreases.

**Figure 2:**
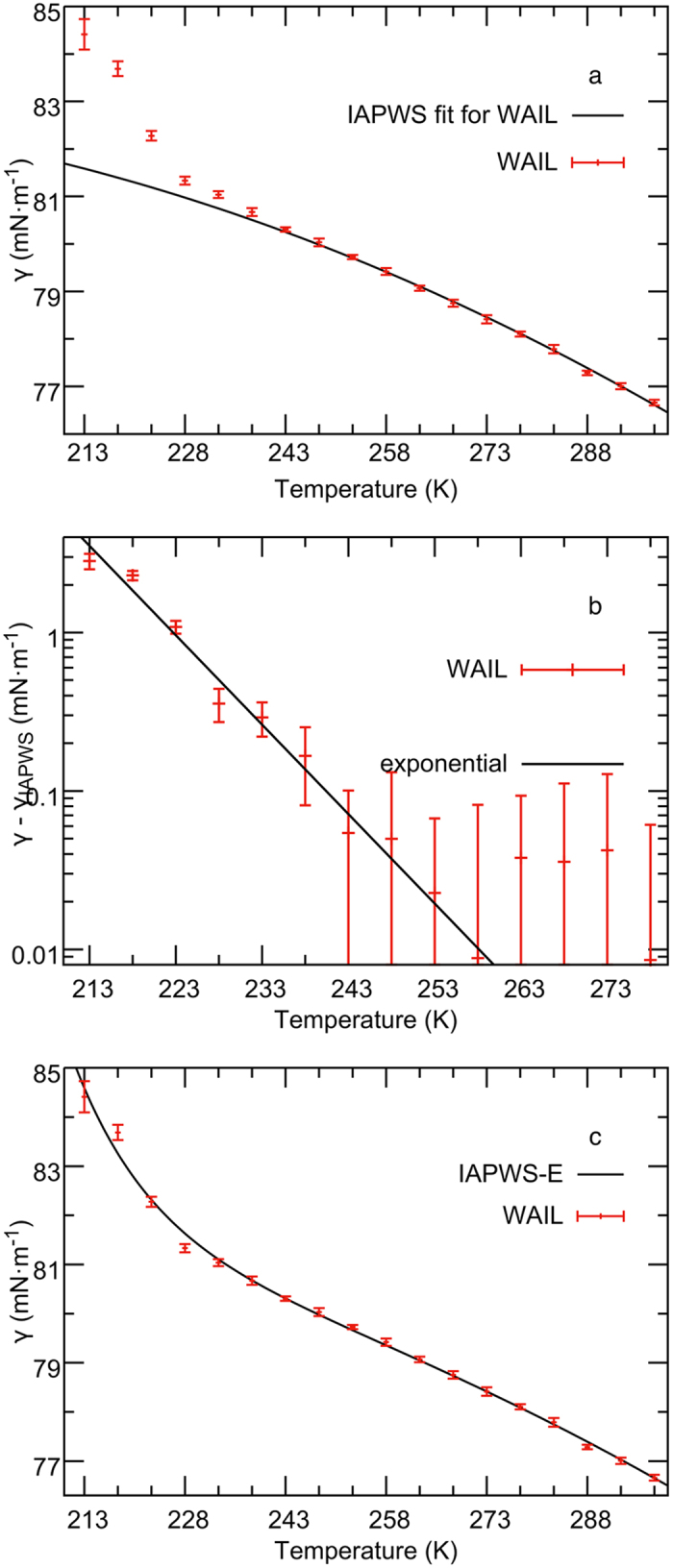
The surface tension of liquid water predicted by the WAIL force field. (**a**) The deviation from the IAPWS equation grows at lower temperatures. (**b**) In a semi-log plot, the deviation is a straight line indicating an exponential growth below 243 K. We note that in (**b**) the deviation above 243 K is so small that the error bar is larger than the signal. Thus, only lower temperature deviations clearly show the exponential behavior. (**c**) The IAPWS-E equation provides a significantly better fit to the WAIL surface tension.

**Figure 3:**
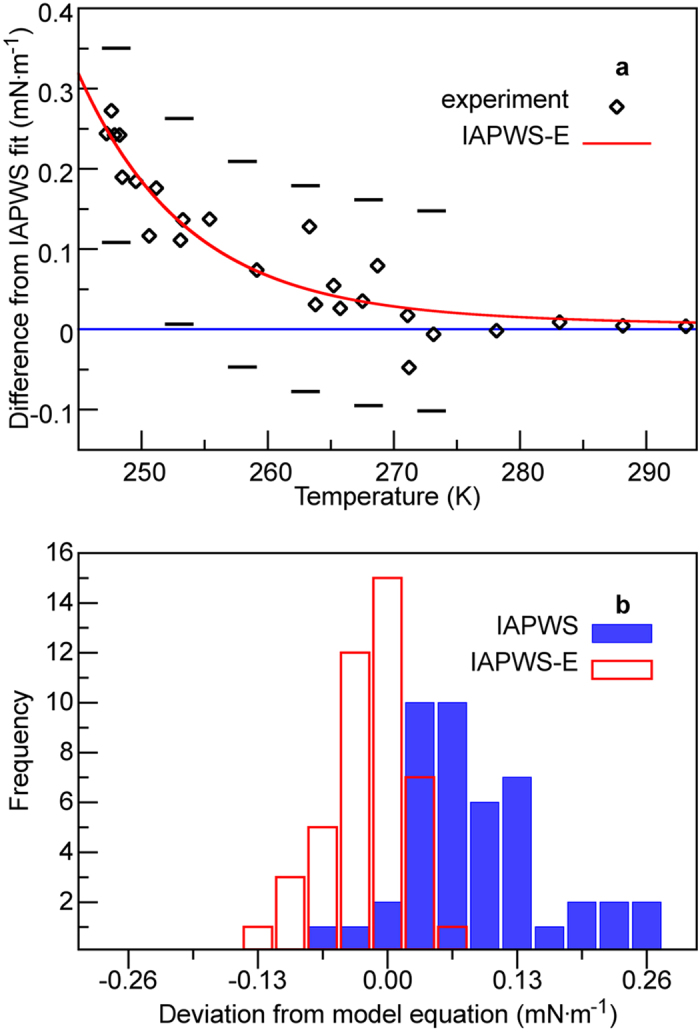
(**a**) The deviation of real water surface tension from the IAPWS equation is captured by the IAPWS-E equation. The experimental error bar shown was reported by Hruby *et al.*^2^. (**b**) The histograms show the distribution of the deviations of the experimental surface tension from the IAPWS or IAPWS-E equations. Although distribution of the deviations from the IAPWS-E equation is symmetric and resembles that of a random noise, the deviations from the IAPWS equation are heavily biased to the positive. Data from all five sets of experimental measurements were included in these histograms.

In Fig. 3, we report the fit using the two datasets obtained by the counter-pressure method. The counter-pressure measurement^2^ has been argued to be more reliable than the classical capillary rise method^1^,^3^,^22^ used for the other datasets. Two additional fits performed with the 2014 and 2015 capillary rise measurements are shown in Figs 1 and 2 in the supplementary information In addition, all five sets of data were fitted together and reported in Fig. 3 in the supplementary information.

When fitting the experiments, we combined supercooled data from 247.23 K to 271.21 K^2^ with the official IAPWS data from 273.16 K to 643.15 K and fixed the *T*_*c*_ to 647.096 K and *μ* to 1.256 as adopted by IAPWS^23^. All other parameters were optimized to minimize the root mean square error of the surface tension. The resulting parameters are summarized in Table 1.

For the counter-pressure datasets, with the IAPWS-E equation, the fitted *B* and *b* are 235.7 mN·m^−1^ and −0.624, respectively, which are in perfect agreement with the 235.8 mN·m^−1^ and −0.625 estimate recommended by IAPWS. The same agreement in *B* and *b* is also observed for the three fits reported in Table 2 in the supplementary information. We note the small difference in the last digit is not caused by the new supercooled data. If the IAPWS equation (2) was fit to the ordinary water surface tension from 273.16 K to 643.15 K reported by IAPWS, we also obtained a *B* of 235.7 mN·m^−1^ and a *b* of −0.624. Thus the small difference is probably due to the slightly different fitting algorithm. Thus, with the exponential term in the IAPWS-E equation, the supercooled data had no effect to the IAPWS parameters up to the last significant number reported. This cannot be said if the IAPWS equation is fit to the full data set from 247.23 K to 647.096 K without the exponential term. In that case, the optimal *B* and *b* are 235.4 mN·m^−1^ and −0.622, respectively. Thus the inclusion of supercooled data changed the IAPWS parameters, leading to larger errors for the well-established ordinary liquid *γ* correlation. We believe this indicates that the supercooled surface tension no longer follows the IAPWS correlation and the deviation can be captured accurately by an exponential term.

It is clear from Fig. 3(a) that the experimental surface tension is well reproduced by the IAPWS-E equation without any signs of systematic deviations at lower temperatures. The distributions of the deviations from the two surface tension fits are shown in Fig. 3(b). Whereas the deviation from the IAPWS equation is biased to the positive, the deviation from the IAPWS-E fit is symmetrical Gaussian shaped that is consistent with a random noise.

It is important to note that the *T*_*e*_ is 234.7 K (Table 1) based on the experimental counter-pressure surface tension. The WAIL potential underestimates the H_2_O ice Ih melting temperature, *T*_*m*_, by 3 K and the D_2_O *T*_*m*_ by 7 K^24^. Since the 270 K *T*_*m*_ of WAIL ice was obtained without considering quantum nuclear effects, we anticipate the WAIL model to under-estimate the ice-Ih *T*_*m*_ by approximately 7-10 K when quantum nuclear effects are accounted for. The 8 K difference between the WAIL *T*_*e*_ and the counter-pressure *T*_*e*_ is consistent with the extent that WAIL underestimates *T*_*m*_. In other words, the extent of supercooling needed to reach *T*_*e*_ is approximately the same for real water and for the WAIL model. Of course, this is probably partially accidental.

For the fits with the two capillary rise datasets reported in the supplementary information, the *T*_*e*_ are 226 K and 228 K, respectively, which is actually in closer agreement with the WAIL prediction in terms of absolute temperature. As reported in the SI, the fit with all five experimental datasets combined gives a *T*_*e*_ of 236 K. We believe a 10 K spread in experimental *T*_*e*_ is expected, considering the relatively large experimental error bars. Such a relatively small variance in *T*_*e*_ from different experimental measurements and from simulations is intriguing.

Nonetheless, it is worth noting the deviation of the experimental data from the IAPWS extrapolation is no larger than 0.3 mN/m even at the lowest temperature. The deviation is so small that it wouldn’t have been possible to establish an exponential signature growing at lower temperatures without the simulations with the WAIL model. There remains the possibility that the positive deviation seen experimentally is a result of some systematic bias present in all data-sets and is unrelated to the exponential feature observed in WAIL water. Further experimental measurements at even lower temperatures will greatly enhance the confidence of the validity of the IAPWS-E fit.

We note that the WAIL potential was developed only based on first principles information and was never fit to any experimental properties. The good agreement between the WAIL and the experimental *T*_*e*_ is not a consequence of some bias that could potentially be introduced when a model was fit to experiments. Assuming the faint exponential signal in the experimental data is the same as the exponential feature that emerges clearly in the WAIL surface tension, it might indicate that the simulated liquid-liquid phase transition in supercooled WAIL water also occurs in real water and is responsible for the exponential growth in the surface tension. The emergence of new physics around 226 K to 235 K revealed by the emerging exponential component in the experimental surface tension is also consistent with the onset of accelerating increase of more structure-ordered metastable water as revealed by X-ray laser measurements at 229 K^25^ in supercooled micro-droplets.

Although that the IAPWS-E equation provides a good description of the WAIL water surface tension down to at least 213 K, we note that the surface tension at even lower temperature will likely deviate from an exponential growth. The exponential growth might be related to an exponential increase of LDL molar faction as temperature decreases. Once the liquid is predominately LDL, the dependence of *γ* on temperature will likely change.

In WAIL water, the exponential growth in surface tension is a direct consequence of approaching the Widom line and the emergence of a new form of water in substantial quantity. Since the surface tension of real water follows a similar behavior as WAIL water with *T*_*e*_ occurring at approximately the same extent of supercooling, we believe that real water is also approaching a Widom line of two different forms of metastable liquids. Although evidence supporting liquid-liquid phase transition has been reported in aqueous solutions^26^ and in confined water^27^,^28^, the small exponential tail in the recent experimental surface tension of supercooled water supports the coexistence of two liquid forms in pure water of macroscopic size.

## Additional Information

**How to cite this article**: Rogers, T. R. *et al.* Possible Evidence for a New Form of Liquid Buried in the Surface Tension of Supercooled Water. *Sci. Rep.* **6**, 33284; doi: 10.1038/srep33284 (2016).

## Supplementary Material

Supplementary Information

## Figures and Tables

**Table 1:** The parameters obtained by fitting the correlation equations to the surface tensions.

Model (data set)	*T*_*c*_ (K)	*μ*	*B* (mN·m^−1^)	*b*	*c* (K^−1^)	*T*_*e*_ (K)
IAPWS2014	647.096	1.256	235.8	−0.625		
IAPWS (WAIL)	711	11/9	206.769	−0.623		
IAPWS-E (WAIL)	711	11/9	209.588	−0.638	0.0857	227.110
IAPWS-E (expt.)	647.096	1.256	235.697	−0.624	0.116	234.669

The experimental parameters released by IAPWS are also reported as a reference and labeled as IAPWS2014 23.*Tc* and *μ* were fixed in all fittings. The experimental data for the IAPWS-E fit were taken from counter-pressure measurements by Hruby *et al.*^2^ for temperatures below 273.16 K, and from the IAPWS release^23^ from 273.16 K to 643.15 K.
