# Supplementary material for: Possible Evidence for a New Form of Liquid Buried in the Surface Tension of Supercooled Water
Source: Sci Rep. 2016 Sep 12;6:33284. doi: 10.1038/srep33284 (PMC5018856; doi:10.1038/srep33284)
Supplement: Supplementary Information [file srep33284-s1.pdf]

Supporting Information for

Possible Evidence for a New Form of Liquid Buried in the Surface Tension of Supercooled Water

T. Ryan Rogers, Kai-Yang Leong, and Feng Wang*
Department of Chemistry and Biochemistry,
University of Arkansas,
Fayetteville, AR 72701

The surface tension of the WAIL water model from 213 to 298 K is reported in Supporting Table 1. The fits to the IAPWS-E equation for the three measurements by the Hruby group using the capillary rise ("height") method is reported in Supporting Table 2 and shown in Supporting Figures 1 and 2. In addition, a fit including all five sets of experimental data is shown in Supporting Figure 3 and also reported in Supporting Table 2. As discussed in the main article, two measurements from the 2015 publication[1] are fit together and labeled as "h 2015". The measurements from the 2014 publication[2] are labeled as "h 2014".

* fengwang@uark.edu

Supporting Table 1. Surface tensions (γ) of WAIL water for the temperature range from 213 K to 298 K.

T (K)	γ (mN/m)
213	84.4±0.3
218	83.7±0.2
223	82.3±0.1
228	81.33±0.08
233	81.04±0.07
238	80.67±0.09
243	80.31±0.05
248	80.03±0.08
253	79.73±0.04
258	79.42±0.07
263	79.07±0.06
268	78.75±0.08
273	78.41±0.09
278	78.10±0.05
283	77.79±0.09
288	77.28±0.04
293	77.01±0.06
298	76.66±0.06

Supporting Table 2. The parameters obtained by fitting the IAPWS-E correlation to experimental surface tension. T_c and μ were fixed in all fittings. In all cases, Hruby's data were only used for temperatures below 273.16 K; the official IAPWS data[3] were used from 273.16 K to 643.15 K. The dataset labeled as "2014+2015" includes data from the 2015 counter-pressure measurements (p 2015) and the 2014 (h 2014) and 2015 (h 2015) capillary rise measurements.

dataset	T_c (K)	μ	B (mN m^{-1})	b	c (K^{-1})	T_e (K)
h 2015	647.096	1.256	235.711	-0.624	0.107	227.557
h 2014	647.096	1.256	235.685	-0.624	0.102	226.399
2014+2015	647.096	1.256	235.682	-0.624	0.140	235.548

Supporting Figure 1. (a) IAPWS-E equation fit to the "h 2015" dataset. (b) Deviation of "h 2015" surface tension from the IAPWS surface tension (black line) is captured by an IAPWS-E equation (blue line). The black bars the experimental error bars estimated by Hruby, et al.[1]

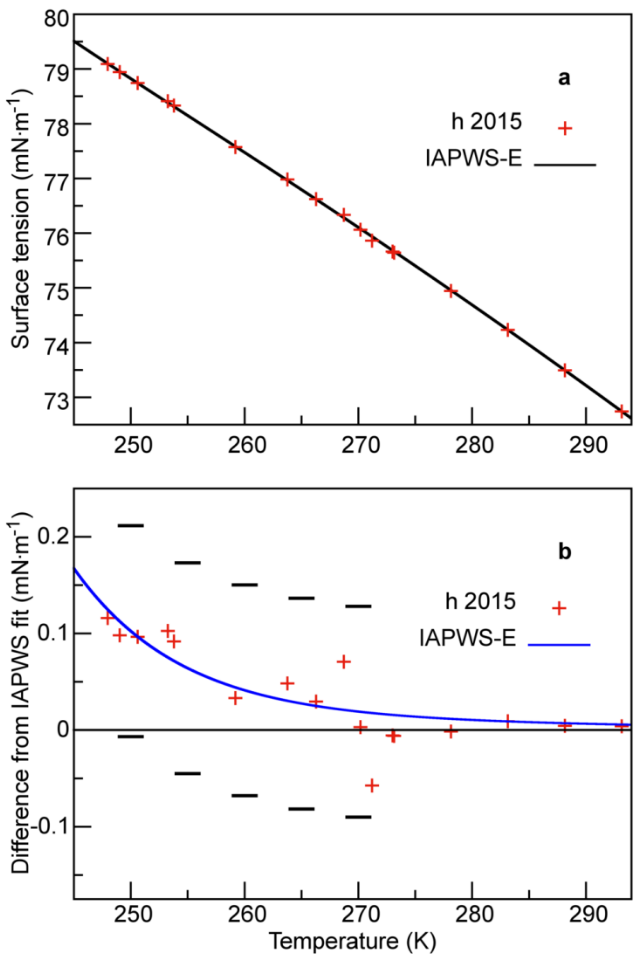

Supporting Figure 2. (a) IAPWS-E equation fit to the “h 2014” dataset. (b) Deviation of “h 2014” surface tension from the IAPWS surface tension (black line) is captured by an IAPWS-E equation (blue line). The black bars the experimental error bars estimated by Hruby, et al.[2]

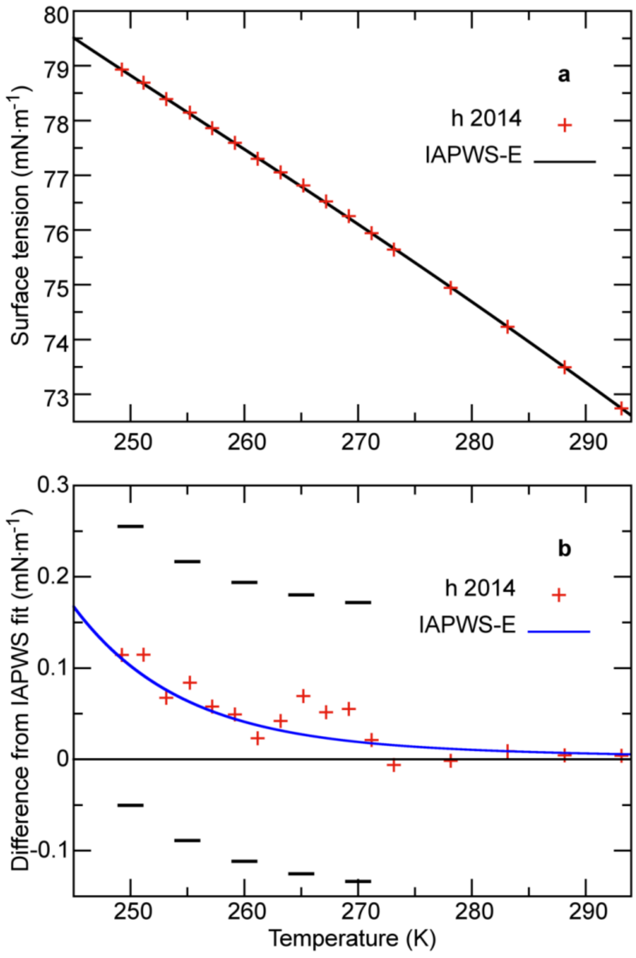

Supporting Figure 3. (a) IAPWS-E equation fit to data from all five datasets (h-1 2015, h-2 2015, p-1 2015, p-2 2015, and h 2014) discussed in the main text. (b) Deviation of the experimental surface tension from the IAPWS surface tension (black line) is captured by an IAPWS-E equation (blue line). The black bars the average of experimental error bars estimated by Hruby, et al.[1,2]

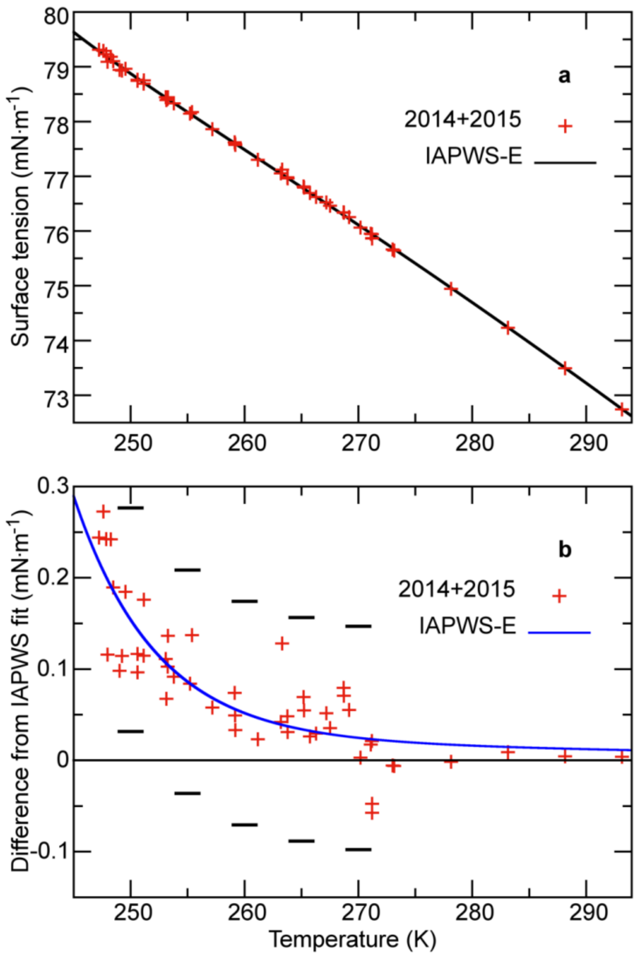

Supporting References

1 Vins, V., Fransen, M., Hykl, J. & Hruby, J. Surface Tension of Supercooled Water Determined by Using a Counterpressure Capillary Rise Method. *J. Phys. Chem. B* **119**, 5567-5575 (2015).

2 Hruby, J., Vins, V., Mares, R., Hykl, J. & Kalova, J. Surface Tension of Supercooled Water: No Inflection Point down to-25 degrees C. *J. Phys. Chem. Lett.* **5**, 425-428, (2014).

3 Dooley, R. B., Revised Release on Surface Tension of Ordinary Water Substance. *International Association for the Properties of Water and Steam* http://www.iapws.org/relguide/Surf-H2O.html (2014) Date of access: 15/07/2015.
